# A nationwide registry study on heart failure in Norway from 2008 to 2018: variations in lookback period affect incidence estimates

**DOI:** 10.1186/s12872-022-02522-y

**Published:** 2022-03-05

**Authors:** Kristina Malene Ødegaard, Sandre Svatun Lirhus, Hans Olav Melberg, Jonas Hallén, Sigrun Halvorsen

**Affiliations:** 1 Institute of Clinical Medicine, University of Oslo, Blindern, P.O. Box 1078, N-0316 Oslo, Norway; 2 Novartis Norway AS, Oslo, Norway; 3 Institute of Health and Society, University of Oslo, Oslo, Norway; 4 Department of Community Medicine, UiT - The Arctic University of Norway, Tromsö, Norway; 5 Arxx Therapeutics, Oslo, Norway; 6 Department of Cardiology, Oslo University Hospital Ulleval, Oslo, Norway

**Keywords:** Heart failure, NPR, Incidence, Prevalence, Wash-out period, Lookback period

## Abstract

**Background:**

The incidence of heart failure (HF) has declined in Europe during the past two decades. However, incidence estimates from registry-based studies may vary, partly because they depend on retrospective searches to exclude previous events. The aim of this study was to assess to what extent different lookback periods (LPs) affect temporal trends in incidence, and to identify the minimal acceptable LP. Further, we wanted to estimate temporal trends in incidence and prevalence of HF in a nationwide population, using the minimal acceptable LP.

**Methods:**

We identified all in- and out-patient contacts for HF in Norway during 2008 to 2018 from the Norwegian Patient Registry. To calculate the influence of varying LP on incident cases, we defined 2018 with 10 years of LP as a reference and calculated the relative difference by using one through 9 years of lookback. Temporal trends in incidence rates were estimated with sensitivity analyses applying varying LPs and different case definitions. Standardised incidence rates and prevalence were calculated by applying direct age- and sex-standardization to the 2013 European Standard Population.

**Results:**

The overestimation of incident cases declined with increasing number of years included in the LP. Compared to a 10-year LP, application of 4, 6, and 8 years resulted in an overestimation of incident cases by 13.5%, 6.2% and 2.3%, respectively. Temporal trends in incidence were affected by the number of years in the LP and whether the LP was fixed or varied. Including all available data mislead to conclusions of declining incidence rates over time due to increasing LPs.

**Conclusions:**

When taking the number of years with available data and HF mortality and morbidity into consideration, we propose that 6 years of fixed lookback is sufficient for identification of incident HF cases. HF incidence rates and prevalence increased from 2014 to 2018.

***Trial registration*:**

Retrospectively registered.

**Supplementary Information:**

The online version contains supplementary material available at 10.1186/s12872-022-02522-y.

## Background

Heart failure (HF) is a chronic disease affecting 1–2% of the adult population worldwide [1]. Both morbidity and mortality are high, making HF a major public health problem with considerable costs [2, 3]. While data from health registries can be used to monitor temporal trends in HF occurrence, the estimates may vary considerably due to different case definitions, data sources and methodological approach [4–7].

Multiple factors such as age distribution, comorbidities, treatment and survival affect the epidemiology of HF. Studies have suggested that HF incidence rates may have been declining during the past 20 years [1] in many European countries, including Sweden [8], Denmark [9] and UK [10]. In Norway, incident hospitalization rates among HF patients declined from 2000 to 2014 [11]. However, recent data suggest that the trend of declining incidence rates is slowing and may be stabilizing [12]. HF prevalence is still increasing due to ageing of the population and improved treatment of cardiovascular diseases and comorbidities [13].

Selection bias occurs in studies based on longitudinal cohorts, since events can occur before the start of the observation period [14]. Incidence estimations from large-scaled health care registries depend on a retrospective search in the database to exclude prior events, termed the lookback period (LP), or sometimes referred to as the wash-out period. Variations in LP will affect the accuracy of the incidence estimates, as misclassifications of prevalent cases as incident cases will decline with increasing lookback length [15–17].

No consensus exists on the minimal length of LP necessary to correctly identify incident HF patient cases from administrative health databases, despite the extensive use of registry data to study disease epidemiology. A short LP will increase misclassification, whereas a long LP will limit the number of reporting years. The primary aims of this study were therefore to assess to what extent different LPs affect incidence estimates of HF, and to identify the minimal acceptable LP utilizing national registry data in Norway. Secondly, we wanted to investigate temporal trends in HF incidence based on the chosen LP.

## Methods

### The Norwegian health care system

The Norwegian healthcare system is publicly funded for all citizens, including hospital admissions and drug treatment. Patients are provided prescription drugs for free except for an annual maximum deductible of 240 USD (2018). The government keep track of health care contacts and expenses with several mandatory national registries. A unique personal 11-digit identification number is used in all contacts with the healthcare system and allows for linking data from different registries, as well as tracking over time.

### Data sources

#### The Norwegian Patient Registry (NPR)

NPR is a nationwide registry covering all hospital contacts and contains diagnoses from all hospital admissions and outpatient consultations, as well as specialist consultations in Norway. All contacts are assigned a primary, and often several secondary, diagnoses according to the 10th revision of the International Classification of Diseases (ICD-10) [18] codes since 2008.

#### The Norwegian Prescription Database (NorPD)

All prescription drugs dispensed to patients are required by law to be registered in the NorPD by the pharmacies. Drugs are registered according to the Anatomical Therapeutic Chemical (ATC) system [19]. Expenses for drugs used in treatment of severe and chronic diseases are reimbursed in Norway. ICD-10 codes and version 2 of the International Classification of Primary Care (ICPC-2) were not included before March 2008 and were fully implemented in March 2009 [20].

#### Statistics Norway

The number, age and sex of all individuals of each calendar year were retrieved from Statistics Norway [21].

### Creation of study population

From NPR, we identified all patients ≥ 18 years of age diagnosed with HF in the period Jan 1, 2008, to Dec 31, 2018. The ICD-10 codes I11.0, I13.0, I13.2, I42.x and I50.x were chosen to identify HF patients. The individual index date was set to the first HF diagnosis in NPR. Data from NPR were subsequently linked to NorPD on an individual level, to collect data on drug dispensations (ATC-codes) and reimbursement codes from both hospitals (ICD-10 codes) and primary care (ICPC-2 codes) to account for comorbidities (see Additional file 1: Table S1 for definitions).

### Definitions of incident HF applying different LPs

An incident HF case was defined as a hospital contact (in-hospital or out-patient) for HF if no previous HF hospital contacts were found retrospectively in the dataset within the LP. We defined 2018 with 10 years of LP as a reference and calculated the relative difference with one through 9 years of lookback to consider the degree of overestimation of incident cases with different lookback lengths.

We calculated temporal trends in incidence by applying both a fixed number of lookback years and including all available data. The following sensitivity analyses were performed:

- 4 years fixed LP (1460 days) from 2012 to 2018,
- 6 years fixed LP (2190 days) from 2014 to 2018,
- 8 years fixed LP (2920 days) from 2016 to 2018, and
- Including all available data and a minimum of 4 years LP from 2012 to 2018

**Fig. 1:**
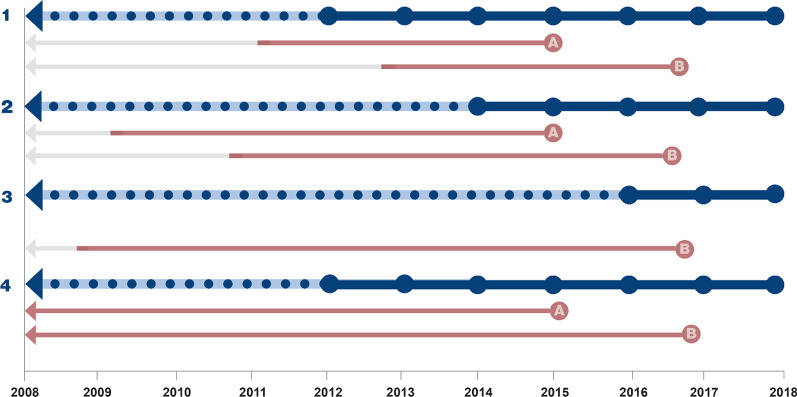
Schematic overview of different lookback approaches. 1; 4 years fixed LP (2012–2018), 2; 6 years fixed LP (2014–2018), 3; 8 years fixed LP (2016–2018); 4, All available data (2012–2018 allowing minimum 4 years of LP resulting in varying LPs). In case examples A and B, the red arrows represent the retrospective lookback period and grey arrows represent deletion of historical data when applying a fixed LP

When applying fixed lookback, historical data prior to the fixed LP was deleted to ensure equal wash-out time for all subjects. Figure 1 shows a schematic overview of the different approaches.

### Definitions of incident HF applying different case definitions

Case definition of HF applied were ICD-10 codes I11.0, I13.0, I13.2, I42.x, or I50.x as a primary or secondary diagnosis. Sensitivity analyses were performed on different case definitions of HF using a 6-year fixed LP from 2014 to 2018:

- Only hospitalized patients with ICD-10 codes I11.0, I13.0, I13.2, I42.x, or I50.x
- ICD-10 codes I11.0, I13.0, I13.2, I42.x, I50.x as primary diagnosis only
- ICD-10 codes I50.x only

Additional file 1: Figure S1 (Supplementary Data) shows the cohort creation flow chart.

### Comorbidities

We identified the following comorbidities for the incident HF population from NPR and NorPD: Atrial fibrillation, cerebrovascular disease, hypertension, ischemic heart disease, myocardial infarction, peripheral artery disease, chronic obstructive pulmonary disease (COPD), anemia, cancer, chronic kidney disease, dementia, depression, diabetes mellitus, dyslipidemia, and thyroid disease. Definitions of comorbidities are listed in the supplementary material.

### Statistical analyses

Categorical variables are presented as frequency distributions and categorical variables as mean or median values with standard deviations or interquartile range. Incidence and prevalence were calculated as crude and age- and sex-specific rates and proportions among the total study population in 2018, including all available data. Temporal trends of incidence and prevalence were calculated as age- and sex-standardised rates and proportions. The population at risk (denominator) for incidence calculations each calendar year was the estimated mid-year population subtracted by the prevalent population in the preceding year (person years). Yearly prevalence was calculated by dividing the total number of prevalent cases each year by the estimated mid-year population. Standardised incidence rates and prevalence were calculated by applying direct age- and sex standardization to the 2013 European Standard Population [22]. Comorbidities and treatment were estimated as proportions among the incidence patient population. Python version 3.X was used for the statistical analysis and data management.

## Results

### Study population

A total of 186,297 patients with the diagnosis of HF were identified in the NPR in the period 2008 to 2018, and 54% were men. The median age was 79 years (interquartile range [IQR] 68–86). Women were older than men (83 years [IQR 74–89] vs. 75 years [IQR 65–84]).

### Impact of lookback period on incident HF cases in 2018

**Fig. 2:**
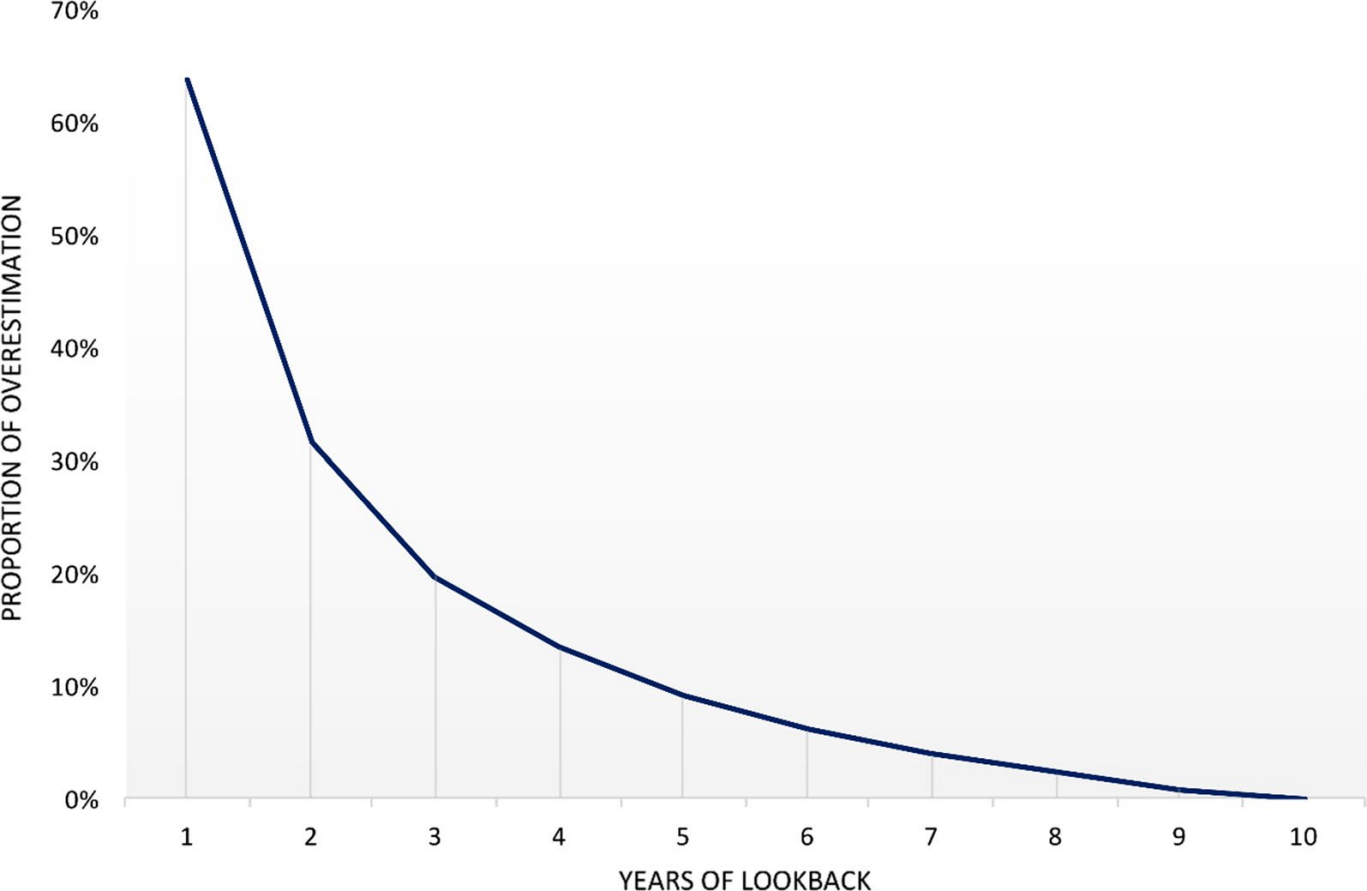
Estimated overestimation (relative difference) of incident HF cases by applying 1–9 years of lookback when using 2018 as year of reference

Using a LP of 10 years, we identified 14,862 incident HF patients in 2018 (6842 women, 8020 men). Table 1 shows how different lengths of LP impacted the number of incident cases and hence incidence rates of HF in 2018 and presents relative difference versus 10 years of lookback. Application of 4, 6 and 8 years of lookback resulted in an overestimation of incident cases by 13.5%, 6.2%, and 2.3%, respectively (11.7%, 5.3%, and 2.0% in women; 15.0%, 7.0%, and 2.6% in men, respectively). The relative difference was higher in men than in women. When stratified by age group and using 6 years of lookback, the relative difference was higher in the age group ≥ 75 years of age being 6.8%, versus 5.3% in patients < 75 years of age (Additional file 1: Table S2). Figure 2 shows that the overestimation of incident cases declined with increasing number of years included in the LP. The overestimation was largest in the beginning of the observational period. For instance, crude incidence rate in 2018 was 3.88 versus 3.74 when using 6 years relative to 8 years of lookback (Table 1).

**Table 1:** Calculations of absolute difference, relative difference, crude and age-standardised incidence rates of HF in 2018 using 1–10 years of lookback

LP (years)	No. of incident cases in 2018	AD, n	RD, %	Crude IR	ASR
*Women*					
10	6842			3.36	3.78
9	6900	58	0.8	3.39	3.81
8	6979	137	2.0	3.43	3.86
7	7076	234	3.4	3.48	3.91
6	7202	360	5.3	3.54	3.98
5	7379	537	7.8	3.63	4.08
4	7642	800	11.7	3.76	4.23
3	8033	1191	17.4	3.95	4.44
2	8751	1909	27.9	4.30	4.84
1	10,548	3706	54.2	5.18	5.82
*Men*					
10	8020			3.95	6.06
9	8116	96	1.2	3.99	6.14
8	8226	206	2.6	4.05	6.23
7	8378	358	4.5	4.12	6.35
6	8578	558	7.0	4.22	6.50
5	8833	813	10.1	4.35	6.71
4	9226	1206	15.0	4.54	7.03
3	9776	1756	21.9	4.81	7.46
2	10,817	2797	34.9	5.32	8.25
1	13,816	5796	72.3	6.80	10.35
*Total*					
10	14,862			3.65	4.74
9	15,016	154	1.0	3.69	4.79
8	15,205	343	2.3	3.74	4.85
7	15,454	592	4.0	3.80	4.94
6	15,780	918	6.2	3.88	5.04
5	16,212	1350	9.1	3.99	5.18
4	16,868	2006	13.5	4.15	5.40
3	17,809	2947	19.8	4.38	5.70
2	19,568	4706	31.7	4.81	6.27
1	24,364	9502	63.9	5.99	7.74

*LP* lookback period, *AD* absolute difference, *RD* relative difference, calculated as the AD divided by the number of incident HF cases for the lookback period of 10 years, expressed as percentage. *IR* incidence rate per 1000 person years, *ASR* age-standardized incidence rate per 1000 person years

### Temporal trends in incidence rate with different LPs

**Fig. 3:**
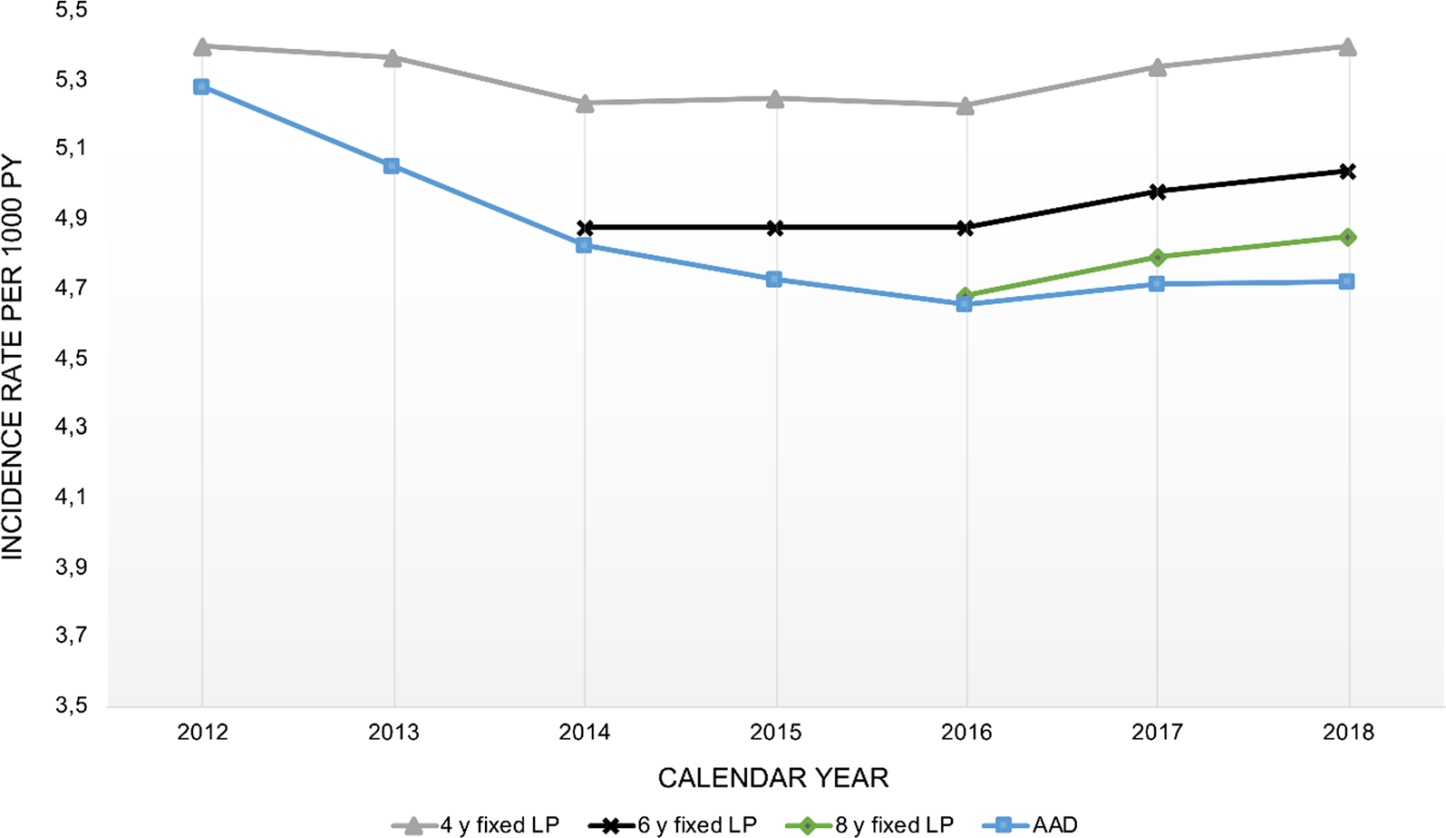
Incidence rates utilizing different approaches to lookback period. Incidence rates are age- and sex standardised per 1000 PY. Grey line; 4 years fixed LP, Black line; 6 years fixed LP, Green line, 8 years LP; Blue line; including all available data from 2014. *PY* person years, *LP* lookback period, *AAD* all available data

For assessment of temporal trends in incidence rate, we performed sensitivity analyses with different lengths of LP (Table 2 and Fig. 3). When applying a fixed number of lookback years, the incidence rates were lower with additional years in the LP. However, we found that the direction and rate of change were similar regardless of using 4, 6 and 8 years of fixed LP. The incidence was stable in the period from 2014 to 2016 with both 4 and 6 years of LP. From 2016 and onwards, the increase in incidence was similar either 4, 6 or 8 years of fixed LP was used. We also performed sensitivity analyses on temporal trends in incidence rate by including all available data, and thereby increasing the LP with time. As expected, incidence rates were lower and declined during the time period when including all available data in contrast to applying a fixed number of lookback years. When including all available data, the incidence rate declined until 2016 from where the incidence rate slightly increased. Figure 3 shows that a relatively shorter LP provided higher incidence estimates and that the direction of the curves was similar when using a fixed LP. Moreover, it shows that including all available data instead of using a fixed LP results in the misleading conclusion of declining incidence rates.

**Table 2:** Incidence and prevalence from 2014 – 2018 with sensitivity analyses

	2014	2015	2016	2017	2018
Incidence rate estimates					
*6 years fixed LP*					
Incident cases, n					
Women	6653	6713	6726	6916	7202
Men	7779	7926	8079	8450	8578
Total	14,432	14,639	14,805	15,366	15,780
Crude incidence rates					
Women	3.40	3.39	3.36	3.43	3.54
Men	3.99	4.01	4.05	4.20	4.22
Total	3.69	3.70	3.71	3.81	3.88
Standardised incidence rates					
Women	3.79	3.80	3.79	3.85	3.98
Men	6.44	6.40	6.39	6.54	6.50
Total	4.88	4.87	4.87	4.98	5.04
*Differences in LP*					
All available data					
Women	3.75	3.69	3.64	3.68	3.76
Men	6.36	6.19	6.07	6.15	6.03
Total	4.83	4.73	4.65	4.71	4.72
4 year fixed LP					
Women	4.04	4.05	4.06	4.11	4.23
Men	6.96	6.95	6.88	7.05	7.03
Total	5.24	5.25	5.23	5.34	5.40
8 year fixed LP					
Women			3.65	3.73	3.86
Men			6.12	6.27	6.23
Total			4.68	4.79	4.85
*Different HF case definitions*^a^					
Only hospitalized^b^					
Women	3.26	3.27	3.31	3.27	3.35
Men	5.48	5.49	5.50	5.56	5.48
Total	4.16	4.18	4.22	4.23	4.25
HF as primary diagnosis^c^					
Women	1.96	2.04	2.03	2.14	2.20
Men	3.48	3.55	3.62	3.78	3.72
Total	2.62	2.70	2.72	2.86	2.86
ICD-10 code I50.x only					
Women	3.53	3.49	3.48	3.53	3.67
Men	6.11	6.11	6.06	6.21	6.17
Total	4.58	4.57	4.54	4.65	4.72
Prevalence estimates					
Prevalent cases, n					
Women	32,581	33,915	35,139	36,458	37,948
Men	41,992	44,653	47,182	49,947	52,684
Total	74,573	78,568	82,321	86,405	90,632
Crude prevalence, %					
Women	1.64%	1.68%	1.73%	1.78%	1.83%
Men	2.11%	2.21%	2.31%	2.42%	2.53%
Total	1.87%	1.95%	2.02%	2.10%	2.18%
Standardised prevalence, %					
Women	1.66%	1.71%	1.76%	1.80%	1.86%
Men	2.93%	3.03%	3.11%	3.20%	3.28%
Total	2.23%	2.30%	2.37%	2.44%	2.51%

Incidence rates are calculated as incident cases per 1000 person-years and presented as age-and sex standardised unless otherwise stated in the table

*LP* lookback period

^a^6 years fixed LP

^b^Excluding outpatient cases

^c^Excluding patients with HF as secondary diagnosis in NPR

### Temporal trends in incidence with different case definitions

**Fig. 4:**
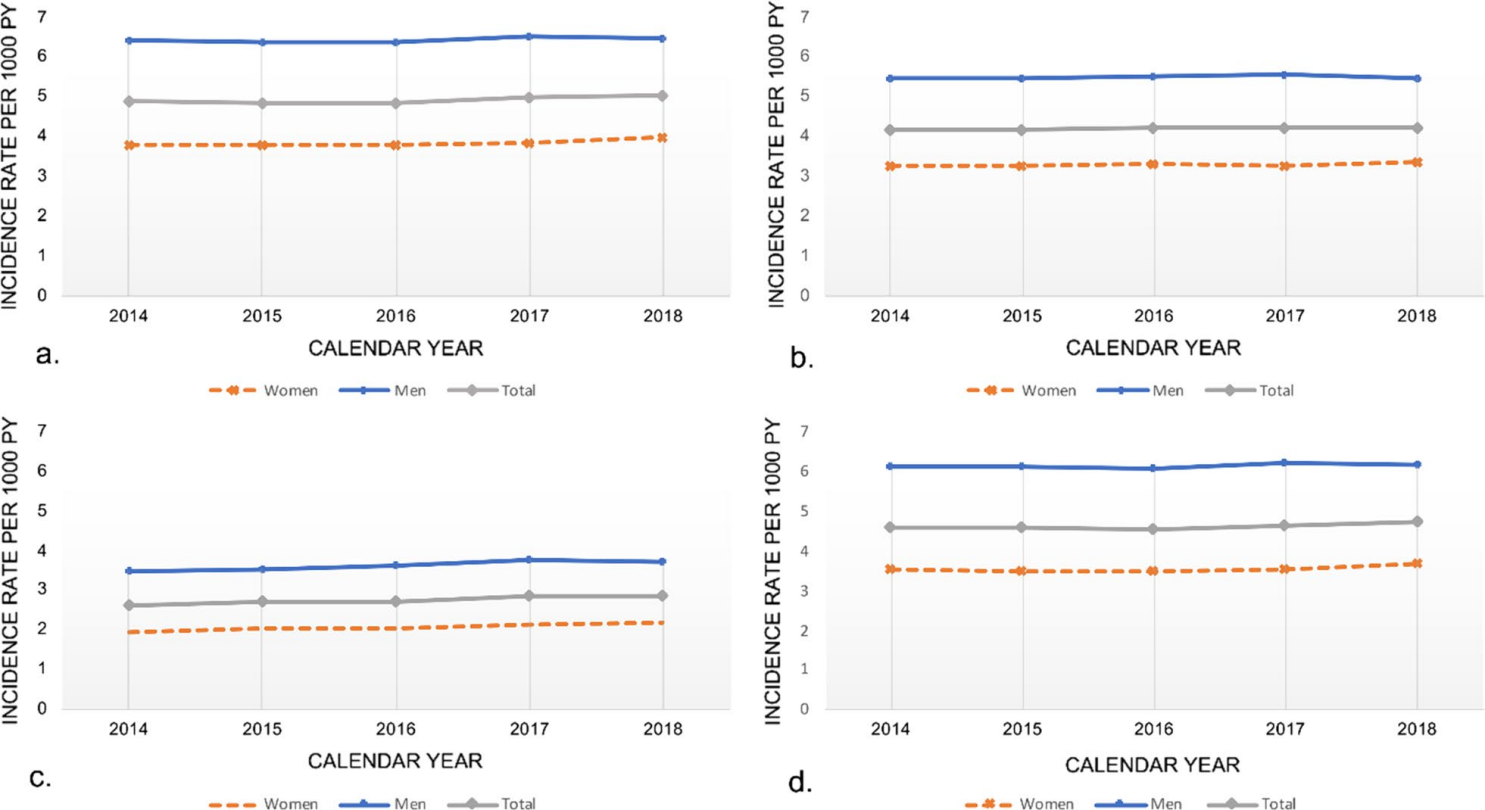
Incidence rates from 2014 to 2018 applying different case definitions of heart failure. Incidence rates are age- and sex standardised per 1000 person-years using 6 years fixed lookback. **a** I11.0, I13.0, I13.2, I42, I50 in any position; **b** Only hospitalized I11.0, I13.0, I13.2, I42, I50, excluding outpatients visit; **c** Only primary diagnosis I11.0, I13.0, I13.2, I42, I50; **d** Only I50.x diagnosis

Similar trends with increasing incidence rates from 2014 to 2018 were shown when performing sensitivity analyses for hospitalized HF patients only (excluding outpatient visits), HF as primary diagnosis (excluding HF as secondary diagnosis) and for ICD-10 code I50 only (excluding I11.0, I13.0, I13.2 and I42). Figure 4 shows the temporal trends in HF incidence from 2014 to 2018 with 6 years of fixed LP when applying different HF case definitions.

### Crude HF incidence and prevalence in 2018

When including all available data (10 years of LP), crude incidence rate in 2018 was 3.64 per 1000 person-years and higher in men than in women (3.93 vs. 3.35 per 1000 person-years, respectively). As expected, incidence rates increased with age, being 76.26 per 1000 person years (68.58 in women and 97.47 in men) in patients ≥ 90 years of age in 2018.

A total of 90,632 patients were classified as prevalent cases in 2018, making the overall crude prevalence 2.2% (2.5% in men and 1.8% in women). Likewise, prevalence increased with age, being 8% (11% in men and 6% in women) in the age group 75–79 and 30% in patients ≥ 90 years of age in 2018 (35% in men and 28% in women). Additional file 1: Table S3 shows the crude incidence rates and prevalence in 2018 by sex and 5-year age groups.

### Temporal trends in standardised incidence and prevalence using 6 years of LP

**Fig. 5:**
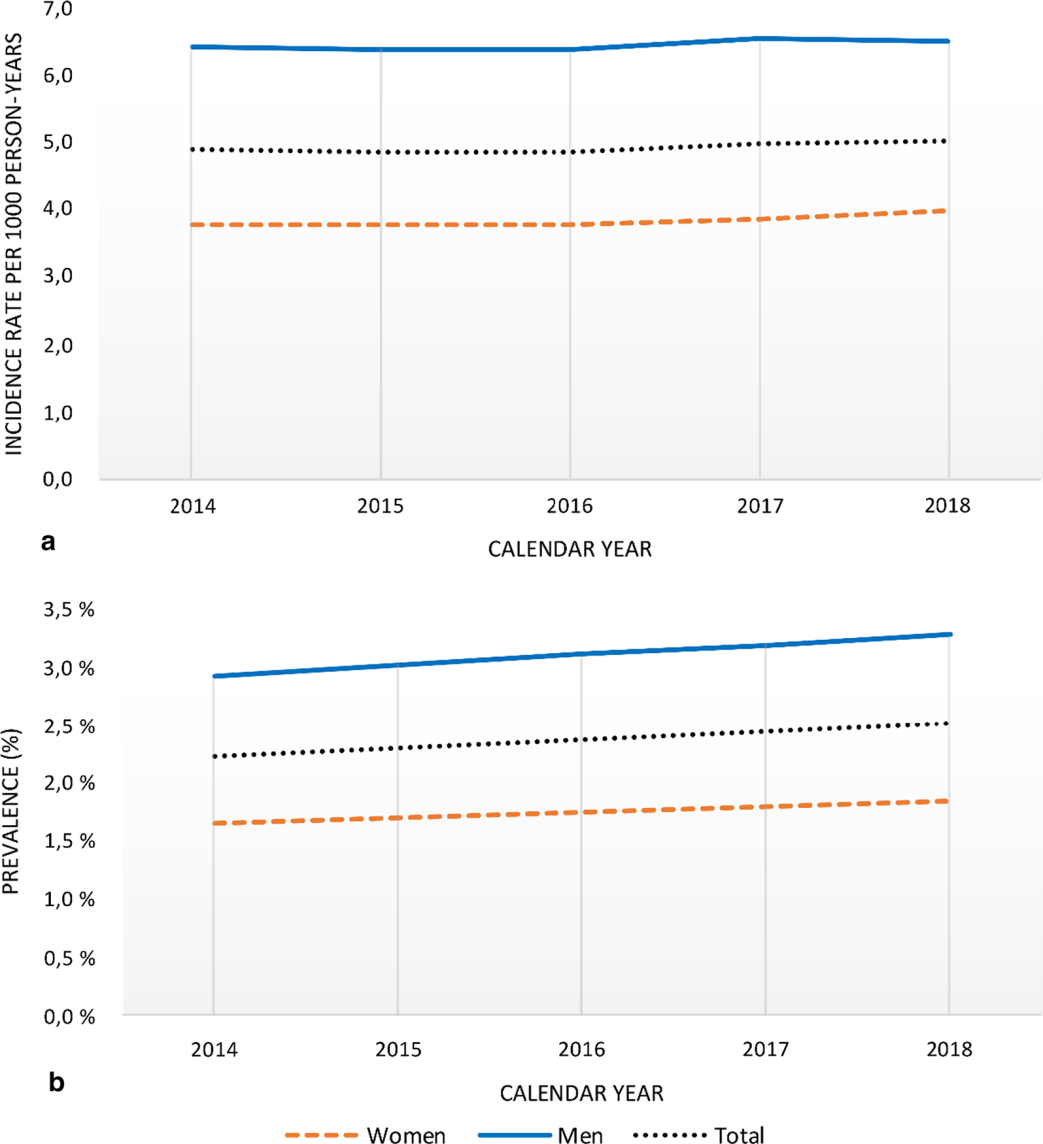
Temporal trends in incidence rates and prevalence from 2014 to 2018 with 6-year fixed lookback period. Age-and sex-standardised **a** incidence rates per 1000 person-years per calendar year and **b** prevalence (%). Blue lines; men, red dashed lines; women, grey; total

When applying 6 years of fixed LP, a total of 75,022 subjects were classified as incident patient cases in the period from 2014 to 2018. Age-and sex-standardised incidence rates increased slightly during the period, being 4.88 per 1000 person-years (95% CI 4.86–4.90) in 2014 and 5.04 per 1000 person-years (95% CI 5.02–5.06) in 2018 (Fig. 5 and Table 2). Incidence rates were higher in men than in women for all age groups. The median age of the total incident HF population during 2014–2018 was 78 years (Interquartile range [IQR] 68–86), and women were older than men (82 [IQR 72–86] and 75 [IQR 65–86], respectively). ICD-10 code I50 was the most common HF-diagnosis (90%). Hypertension was the most common comorbidity (69%), followed by dyslipidemia (54%), atrial fibrillation (48%) and ischemic heart disease (46%). Table 3 presents baseline characteristics for the incident population by calendar year.

**Table 3:** Characteristics of incident HF patients from 2014 to 2018 by calendar year

	Total	2014	2015	2016	2017	2018
No. of cases	75,022	14,432	14,639	14,805	15,366	15,780
Male gender						
n	40,812	7779	7926	8079	8450	8578
%	54%	54%	54%	55%	55%	54%
Age						
Mean (SD)	75.8 (14.1)	76.0 (14.1)	75.7 (14.2)	75.8 (14.2)	75.6 (14.2)	75.7 (14.1)
Median (IQR)	78 (68–86)	79 (68–87)	78 (68–86)	78 (68–87)	78 (68–86)	78 (68–86)
Age groups (%)						
< 75	40%	39%	40%	41%	41%	41%
≥ 75	60%	61%	60%	59%	59%	59%
≥ 85	32%	32%	32%	32%	31%	30%
ICD-10 codes						
I11.0	3%	3%	3%	4%	3%	3%
I13.0	0%	0%	0%	0%	0%	0%
I13.2	0%	0%	0%	0%	0%	0%
I42.x	9%	9%	9%	9%	10%	10%
I50.x	90%	90%	90%	89%	89%	90%
I50.0	13%	13%	13%	13%	13%	14%
I50.1	19%	17%	19%	20%	20%	21%
I50.9	57%	60%	58%	57%	56%	55%
Comorbidities^a^						
Cardiovascular						
Atrial fibrillation	48%	47%	47%	48%	48%	49%
Cerebrovascular event	16%	16%	16%	15%	16%	16%
Hypertension^b^	69%	69%	69%	69%	68%	69%
Ischemic heart disease	46%	49%	47%	46%	45%	44%
Myocardial infarction	24%	25%	24%	23%	23%	22%
Peripheral artery disease	15%	14%	15%	14%	15%	15%
Other						
COPD	22%	21%	22%	22%	22%	23%
Anemia	24%	23%	24%	24%	24%	24%
Cancer	22%	21%	22%	22%	22%	23%
Chronic kidney disease	23%	22%	22%	23%	24%	24%
Dementia	6%	6%	6%	6%	5%	5%
Depression	22%	22%	22%	23%	21%	22%
Diabetes mellitus	21%	21%	21%	21%	21%	21%
Dyslipidemia	54%	53%	54%	54%	55%	56%
Thyroid disease	11%	11%	11%	11%	11%	12%

Incident patients are defined as first HF contact with 6 years of fixed lookback. Data are presented as frequencies (%), median (IQR) or mean (SD)

*SD* standard deviation, *IQR* interquartile range

^a^0–2190 days before index

^b^180–2160 days before index

Standardised prevalence increased from 2.23 to 2.51% during 2014 to 2018, equivalent to a 22% increase in the absolute patient population. The prevalence was higher in men than in women, consistent in all age groups (Additional file 1: Figure S2).

## Discussion

This study examined the effects of varying LP on the incidence of HF, and calculated incidence rate and prevalence from 2014 to 2018. Firstly, we found that compared to a 10-year LP, 4 to 8 years of LP overestimated the incidence by 13.5% to 2.3%. Secondly, we found that the incidence increased from 2014 to 2018 when using a fixed LP regardless of HF case definition applied.

Registry based studies of HF have used different length of LPs for calculation of incidence estimates. These differences make interpretation and comparison of studies difficult. The extent of misclassification depends on the nature of the respective disease. Previous studies have investigated the impact of varying LP for different disease areas, including stroke [23, 24], coronary heart disease [25], diabetes and cancer [17]. Other studies have identified optimal lookback length to assess prevalence of comorbidities [24, 26]. Recently, a report on Swedish registries illustrated how short LPs cause overestimation of the incidence rates of multiple chronic diseases, excluding HF [27]. Few studies address and suggest the minimum lookback length for specific diseases. For acute myocardial infarction (AMI), LPs of 7–10 years have been considered reliable to identify incident events on individual level in NPR [16]. Regarding HF, there are scarce data on the impact of different LPs on the incidence estimates. American reports of HF incidence rates, mainly based on Medicare claims, have commonly been using a 1 year or shorter LP [28–30], although this LP is reported too short to avoid misclassification [31]. Camplain et al. [32] found that shorter LP overestimated incidence rates compared to 3 years. Our results indicate that 3 years of LP would overestimate incidence rate by 19.8% in our study. A study based on a statutory health insurance sample in Germany from 2000 to 2008 found an overestimation of 42.7%, 24.2 and 4.8% when applying 1, 2 and 5 years of lookback, respectively, relative to 8 years of lookback [17]. Our results suggest that even 8 years of lookback is not enough to correctly discriminate between incident and prevalent HF cases.

The length of the LP constitutes a tradeoff between the number of reported years and the accuracy of the estimate, as some available data is ignored when using fixed lookback. The most common approach has been to use a fixed LP instead of including all available data and thus varying LPs when assessing temporal trends in incidence, despite the risk of overestimating incidence [33]. This is because even though inclusion of all available data will optimize the accuracy of the estimates for any given year, the trend in incidence over time will be biased due to the variability of the length of LP [34, 35]. Consistent with this, we found that when the individual LP was maximised for each patient the trend line suggested a declining incidence over time, compared to a fixed LP. This is explained by the increased number of patients classified as prevalent as more years were included in the LP. The decrease is highest in the beginning, corresponding to a higher impact of overestimation the first years. The increase in incidence rate from 2016 onwards was, on the contrary, smaller compared to applying fixed number of lookback years. The trend line flattens out from 2016 due to smaller impact of additional lookback years in combination with an underlying increase in incidence rate. Including all available data will therefore most likely underestimate the increase in incidence rate from 2016 to 2018. While the risk of misclassifying incident cases diminishes for each year when including all available data, a fixed LP approach generates a fair comparison of incidence rates between calendar years when assessing temporal trends.

In our study, we calculated the relative difference with different lengths of lookback by using 2018 as a year of reference. As the acceptable extent of overestimation is rather arbitrary, the decision on minimum acceptable lookback years needs to be considered in light of several important aspects. Number of years with available data, disease characteristics, population age, fatality rates, and time trends in survival and recurrence rates are key factors [16]. Firstly, eleven calendar years of individual data from NPR were available from 2008 to 2018, limiting our possibilities of longer LPs than 10 years. The mean age at first HF diagnosis is relatively high, representing a higher likelihood of having a previous hospital contact when compared to a younger patient population. This suggests the need of a relatively longer LP. However, several factors suggest that a shorter LP might be sufficient. The high mortality and morbidity associated with HF suggest that a shorter LP may be used to identify the incident event. Data from the ESC-HF-LT registry reported 1-year all-cause mortality rates of 23.6% for acute HF patients and 6.4% for chronic HF patients between 2011 and 2013 [36] and 5-year mortality rates are reported to be as high as ≥ 50% [5, 37]. Morbidity is also high, with acute HF being the primary cause of hospitalization in patient > 65 years of age [38]. Every fourth Norwegian HF patient > 67 years of age is readmitted within 30 days after hospitalization [39]. According to the ESC-HF pilot study published in 2013, 1 year hospitalization rates were reported to be 44% in hospitalized patients and 32% in ambulatory patients [40]. Altogether, we considered 6 years of lookback as sufficient for calculating incidence rates, given the number of available years from our dataset. When merely assessing the direction of temporal trend lines, application of even shorter LP may be considered.

We found that temporal trends in incidence rates increased from 2014 to 2018. The increase was greatest from 2016 onwards. This contrasts with previous reports from Europe [1, 8–10]. To our knowledge, no recent studies have shown a similar trend. A study from our group based on prescription data from NorPD showed an unchanged incidence rate of HF in the period from 2013 to 2016 [12]. These two studies are not directly comparable due to different data sources and inclusion criteria. However, the former study included all available data when estimating temporal trends in incidence, possibly underestimating a true increase in incidence rate. The Norwegian CVDNOR project showed a decrease in incident HF hospitalizations as primary discharge diagnosis from 2000 to 2014 [11]. That study is to a large extent comparable to our analysis, as they used 6 years of fixed lookback. In our study, incidence rates increased from 2014 to 2018 also when applying different case definitions as sensitivity analyses. Even though our study was not designed to investigate reasons for a possible shift in temporal trends of HF incident rates in this study, the increase in incidence from 2016 may partly be a result of improved diagnostic practices, after the introduction of a new therapeutic drug class in the 2016 European HF guidelines [1] and increased attention towards HF patients. This remains speculative.

We found an increase in prevalence throughout the study period of 2014–2018, consistent with previous findings. Despite advances in HF management and treatment of comorbidities resulting in better survival [1, 36], the ageing of the population most likely explains the majority of the increase in prevalence.

### Strengths and limitations

A strength of this study is the use of the NPR database with complete coverage of all national HF contacts, including both hospitalizations and outpatient visits. The Norwegian health care system is publicly funded, and insurance policies do not influence management of HF. Linkage with NorPD allows for the inclusion of additional information on important comorbidities and treatment following discharge.

Some important limitations need to be addressed with regards to data quality and study design. Firstly, the inclusion criterion for HF was a case definition based on ICD-10 codes, which to date is not yet validated in Norway. However, validation studies of ICD-10 codes in NPR have demonstrated high quality for research purposes, including several cardiovascular diseases [41–44]. Validation studies from other countries have shown that specificity and positive predictive value for HF is high but with lower sensitivity [45]. This is also the case within the Nordic countries; however, evidence is more conflicting [46–50]. NPR does not include individuals without any contact with specialist care during the study period, representing a potential selection bias. The reimbursement model for health care services in Norway may influence the use of ICD-10 codes and might change over time.

Secondly, the relative short time period of observations and the application of 10 years of lookback in 2018 for identifying incident cases may represent a limitation considering lacking a validation study. Nonetheless, alternatives for correctly identifying incidence cases would include a longer observational period, access to electronic medical records or self-reported data, neither available in this study. We considered 10 years of LP to be sufficient as a reference considering HF is a chronic disease with high morbidity and mortality.

Differences in study populations, time periods and case definition make comparison between studies difficult, but our results will most probably be pertinent for calculating HF incidence also in other HF populations.

## Conclusions

Incidence rates of HF from register-based studies vary with different lengths of LP. Trade-offs need to be made between the length of the LP and years of data presented, considering the number of years with available data and high HF mortality and morbidity. We propose that 6 years of fixed lookback is sufficient for identification of incident HF cases. HF incidence rates increased from 2014 to 2018, in contrast with previous reports up until 2014.

## Supplementary Information

**Additional file 1:** Supplementary Data.

## Data Availability

The data that support the findings of this study are available from the Norwegian Directorate of Health (https://helsedata.no/en/) and The Norwegian Institute of Public Health (NIPH) (https://www.fhi.no/en/hn/health-registries/norpd/Access-data-norpd/). Access is granted to researchers, but it is subject to regulations and requires permissions from the Norwegian Directorate of Health and NIPH. Data are available from the corresponding author upon reasonable request if permissions are granted.

## Abbreviations

HF: Heart failure
LP: Lookback period
NorPD: Norwegian Prescription Database
NPR: National Patient Registry
