# Supplementary material for: A nationwide registry study on heart failure in Norway from 2008 to 2018: variations in lookback period affect incidence estimates
Source: BMC Cardiovasc Disord. 2022 Mar 5;22:88. doi: 10.1186/s12872-022-02522-y (PMC8898410; doi:10.1186/s12872-022-02522-y)

Supplementary Data

A nationwide registry study on heart failure in Norway from 2008-2018: variations in lookback period affects incidence estimates

Kristina Malene Ødegaard, Sandre Svatun Lirhus, Hans Olav Melberg, Jonas Hallén, and Sigrun Halvorsen

Table of Contents

Table S1. Definitions 2

Figure S1. Cohort creation flow chart 4

Table S2. The number of incident heart failure cases detected by various length of lookback periods (1 to 10 years) and relative difference by calendar year (2009-2018), by age group 5

Table S3. Crude incidence and prevalence of HF in 2018, stratified by sex and age group 6

Figure S2. Yearly prevalence from 2014-2018 by 5-year age groups 8

Table S1. Definitions

**Condition**	**NPR: ICD-10 code or procedure code (NOMESCO)**	**NorPD: ATC code or reimbursement code (ICD-10 or ICPC-2)**
Heart Failure	I11.0, I13.0, I13.2, I42.x, I50.x	
Atrial fibrillation	I48	Reimbursement codes: I48, K78 (ICPC)
Cerebrovascular disease (including stroke and TIA)	G45, G46, H34.0, I60-I69	
Hypertension (at least 180 days prior to HF index)	I10, I11, I12, I13, I15	Reimbursement codes: I10-I13, I15 (ICD10) or K86, K87 (ICPC)
Ischemic heart disease	I20, I21, I22, I23, I24, I25	Reimbursement codes I20-I25 (ICD-10)
Myocardial infarction	I21, I22	
Peripheral artery disease	I70, I71, I72, I73, I74, I77, I78, I79, R02	
COPD	J40-J44	Reimbursement codes J40-J44 (ICD-10) or R95 (ICPC-2)
Anemia	D50, D51, D52, D53, D55, D56, D57, D58, D59, D60, D61, D62, D63,D64	ICPC: Reimbursement codes B80, B81, B82 or ICD10: D50, D51, D52, D53, D55, D56, D57, D58, D59, D60, D61, D62, D63,D64
Cancer	C00, C01, C02, C03, C04, C05, C06, C07, C08, C09, C10, C11, C12, C13, C14, C15, C16, C17, C18, C19, C20, C21, C22, C23, C24, C25, C26, C30, C31, C32, C33, C34, C37, C38, C39, C40, C41, C43, C44, C45, C46, C47, C48, C49, C50,	

	C51, C52, C53, C54, C55, C56, C57, C58, C60, C61, C62, C63, C64, C65, C66, C67, C68, C69, C70, C71, C72, C73, C74, C75, C76, C77, C78, C79, C80, C81, C82, C83, C84, C85, C86, C88, C90, C91, C92, C93, C94, C95, C96, C97	
Chronic kidney disease	N17-N19	
Dementia	F00, F01, F02, F03, G30, G31.8	Reimbursement code P70 (ICPC)
Depression	F32, F33	Reimbursement codes P03 (ICPC), P76 (ICPC)<br>Or ATC-code N06A
Diabetes Mellitus	E10-E14	ATC codes: A10A or A10B<br>or reimbursement codes: E10, E11, E13, E14 (ICD-10)<br>or T89, T90 (ICPC-2)
Dyslipidemia	E78	Reimbursement codes: E78 (ICD-10)<br>or T93 (ICPC)<br>ATC-codes:C10A or C10B
Thyroid disease		ATC-codes: H03, H01AB or Reimbursement codes T85 (ICPC), T86 (ICPC)

Figure S1. Cohort creation flow chart

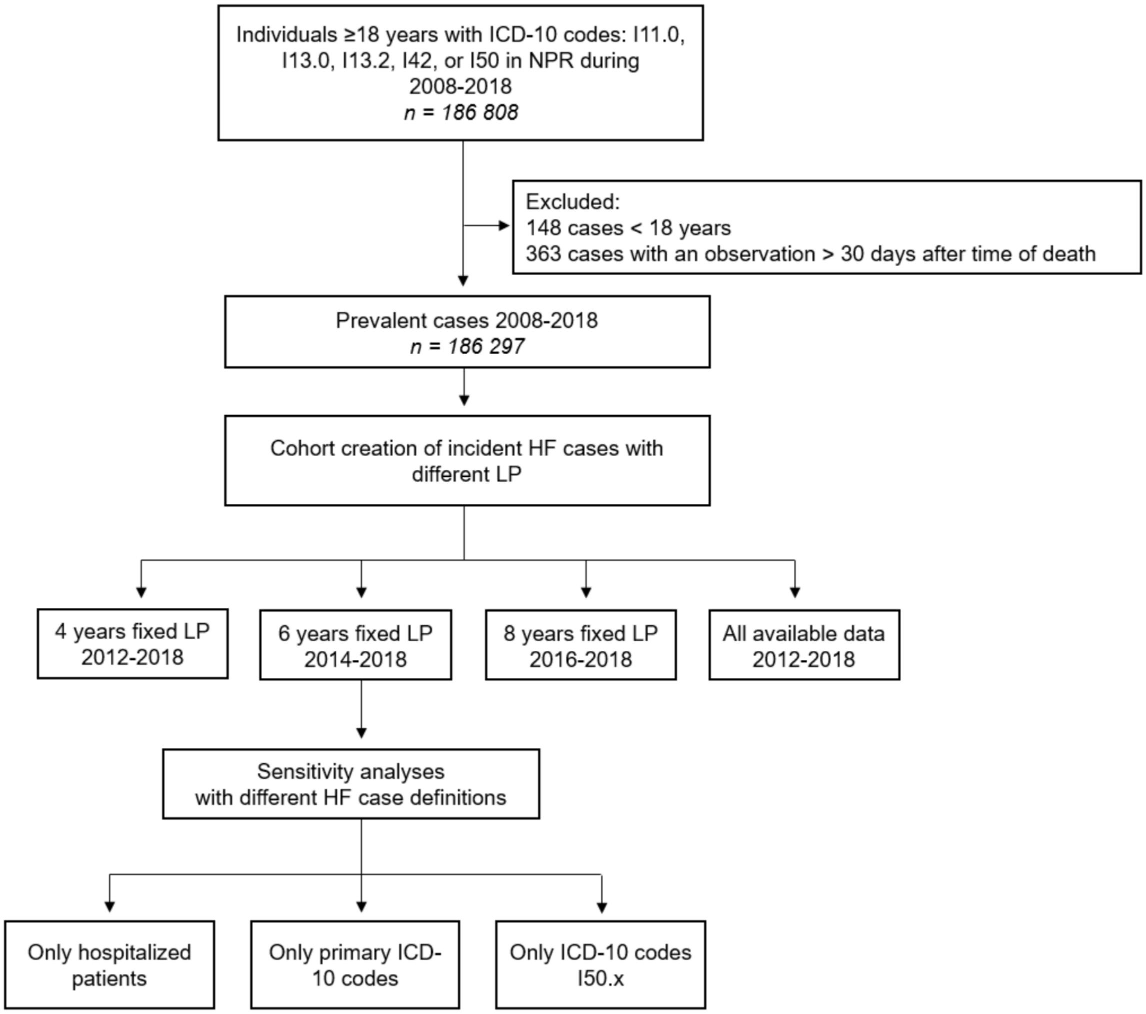

NPR, Norwegian Patient Registry; LP, Lookback Period; HF, Heart Failure

Table S2. The number of incident heart failure cases detected by various length of lookback periods (1 to 10 years) and relative difference by calendar year (2009-2018), by age group

												**2018**	
**Calendar year**	2009	2010	2011	2012	2013	2014	2015	2016	2017	2018	LP	**AD, n**	**RD, %**
**Age group**													
**> 75**	14891	14767	13900	14270	14110	13691	13818	13708	13864	14149	1	5379	61.3 %
		12102	11412	11701	11562	11239	11162	11260	11461	11725	2	2955	33.7 %
			10157	10567	10374	10133	10060	10198	10364	10673	3	1903	21.7 %
				9858	9708	9518	9469	9502	9744	10077	4	1307	14.9 %
					9246	9084	9060	9097	9339	9643	5	873	10.0 %
						8811	8749	8806	9040	9365	6	595	6.8 %
							8535	8582	8825	9154	7	384	4.4 %
								8420	8681	8995	8	225	2.6 %
									8572	8874	9	104	1.2 %
										8770	10		
**<75**	7874	8203	8164	8460	8855	8808	9314	9510	9901	10215	1	4123	67.7 %
		6612	6482	6765	6890	6889	7247	7422	7756	7843	2	1751	28.7 %
			5916	6140	6266	6242	6582	6721	7043	7136	3	1044	17.1 %
				5821	5968	5935	6255	6360	6711	6791	4	699	11.5 %
					5785	5746	6042	6155	6493	6569	5	477	7.8 %
						5621	5890	5999	6326	6415	6	323	5.3 %
							5780	5889	6221	6300	7	208	3.4 %
								5810	6132	6210	8	118	1.9 %
									6072	6142	9	50	0.8 %
										6092	10		

Table S3. Crude incidence and prevalence of HF in 2018, stratified by sex and age group

	Prevalence			Incidence		
	**N**	**n**	**% (95% CI)**	**PY**	**n**	**IR (95% CI)**
**Total**	4153010.5	90632	2.18 (2.17 -2.20)	4066606	14796	3.64 (3.58-3.70)
**Sex**						
Women	2071252.5	37948	1.83 (1.81-1.85)	2034795	6814	3.35 (3.27-3.43)
Men	2081758	52684	2.53 (2.51-2.55)	2031811	7982	3.93 (3.84-4.01)
**By age group, Women**						
18-24	230064	92	0.04 (0.03-0.05)	229975	20	0.09 (0.05-0.13)
25-29	183206	179	0.10 (0.08-0.11)	183021	27	0.15 (0.09-0.20)
30-34	175145.5	272	0.16 (0.14-0.17)	174909	39	0.22 (0.15-0.29)
35-39	169989	316	0.19 (0.17-0.21)	169721	53	0.31 (0.23-0.40)
40-44	172466	405	0.23 (0.21-0.26)	172072	58	0.34 (0.25-0.42)
45-49	185550	670	0.36 (0.33-0.39)	184896	95	0.51 (0.41-0.62)
50-54	174390.5	931	0.53 (0.50-0.57)	173546	142	0.82 (0.68-0.95)
55-59	157569	1219	0.77 (0.73-0.82)	156431	196	1.25 (1.08-1.43)
60-64	149228.5	1767	1.18 (1.13-1.24)	147572	285	1.93 (1.71-2.16)
65-69	135468.5	2669	1.97 (1.90-2.04)	132923	464	3.49 (3.17-2.81)
70-74	124944	4431	3.55 (3.44-3.65)	120812	782	6.47 (6.02-6.93)
75-79	82329	4855	5.90 (5.74-6.06)	77734	880	11.32 (10.58-12.06)
80-84	59079.5	5762	9.75 (9.51-9.99)	53696	1076	20.04 (18.85-21.22)
85-89	43142	6599	15.30 (14.96-15.64)	36403	1251	34.37 (32.49-36.24)
≥ 90	28681	7781	27.13 (26.61-27.64)	21086	1446	68.58 (65.17-71.99)
**By age group, Men**						
18-24	246372	151	0.06 (0.05-0.07)	246229	46	0.19 (10.13-0.24)
25-29	190617.5	246	0.13 (0.11-0.15)	190379	30	0.16 (0.10-0.21)
30-34	184118	351	0.19 (0.17-0.21)	183782	44	0.24 (0.17-0.31)
35-39	180407	513	0.28 (0.26-0.31)	179912	69	0.38 (0.29-0.47)
40-44	182129.5	735	0.40 (0.37-0.43)	181453	109	0.60 (0.49-0.71)
45-49	195061.5	1327	0.68 (0.64-0.72)	193774	227	1.17 (1.02-1.32)
50-54	183787.5	2338	1.27 (1.22-1.32)	181600	329	1.81 (1.62-2.01)
55-59	163937.5	3306	2.02 (1.95-2.08)	160717	446	2.78 (2.52-3.03)
60-64	149878.5	4857	3.24 (3.15-3.33)	145262	647	4.45 (4.11-4.80)
65-69	135216.5	6279	4.64 (4.53-4.76)	129193	831	6.43 (6.00-6.87)
70-74	119042.5	8594	7.22 (7.07-7.37)	110962	1129	10.17 (9.58-10.77)

75-79	70758.5	7672	10.84 (10.61-11.07)	63687	1145	17.98 (16.95-19.01)
80-84	43691	6759	15.47 (15.13-15.81)	37408	1170	31.28 (29.51-33.04)
85-89	25375	5631	22.19 (21.68-22.70)	19814	1015	51.23 (48.16-54.30)
≥ 90	11365.5	3925	34.53 (33.66-35.41)	7644	745	97.47 (90.82-104.12)
**By age group, Total**						
18-24	476436	243	0.05 (0.04-0.06)	476204	66	0.14 (0.11-0.17)
25-29	373823.5	425	0.11 (0.10-0.12)	373400	57	0.15 (0.11-0.19)
30-34	359263.5	623	0.17 (0.16-0.19)	358691	83	0.23 (0.18-0.28)
35-39	350396	829	0.24 (0.22-0.25)	349633	122	0.35 (0.29-0.41)
40-44	354595.5	1140	0.32 (0.30-0.34)	353525	167	0.47 (0.40-0.54)
45-49	380611.5	1997	0.52 (0.50-0.55)	378670	322	0.85 (0.76-0.94)
50-54	358178	3269	0.91 (0.88-0.94)	355145	471	1.33 (1.21-1.45)
55-59	321506.5	4525	1.41 (1.37-1.45)	317148	642	2.02 (1.87-2.18)
60-64	299107	6624	2.21 (2.16-2.27)	292833	932	3.18 (2.98-3.39)
65-69	270685	8948	3.31 (3.24-3.37)	262115	1295	4.49 (4.67-5.21)
70-74	243986.5	13025	5.34 (5.25-5.43)	231774	1911	8.25 (7.88-8.61)
75-79	153087.5	12527	8.18 (8.05-8.32)	141421	2025	14.32 (13.70-14.94)
80-84	102770.5	12521	12.18 (11.98-12.38)	91104	2246	24.65 (23.65-25.66)
85-89	68517	12230	17.85 (17.56-18.14)	56217	2266	40.31 (38.68-41.93)
≥ 90	40046.5	11706	29.23 (28.79-29.68)	28730	2191	76.26 (73.19-79.33)

Unadjusted prevalence and incidence in 2018 utilizing all available data. CI; Confidence intervals. PY; person years. IR; Incidence rate per 1000 person years.

Figure S2. Yearly prevalence from 2014-2018 by 5-year age groups

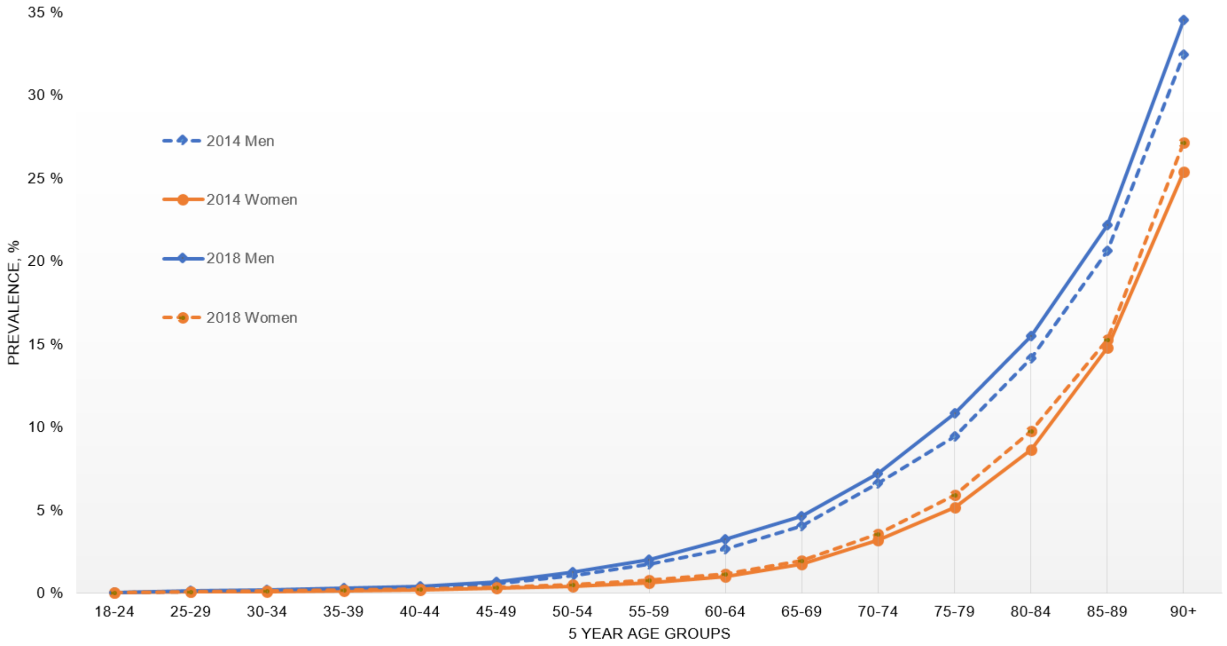

Supplement: Supplementary file 1 — Additional file 1: Supplementary Data. [file 12872_2022_2522_MOESM1_ESM.pdf]
